# Supplementary material for: Targeted High-Throughput Sequencing Enables the Detection of Single Nucleotide Variations in CRISPR/Cas9 Gene-Edited Organisms
Source: Foods. 2023 Jan 18;12(3):455. doi: 10.3390/foods12030455 (PMC9914749; doi:10.3390/foods12030455)
Supplement: Supplementary file 1 [file foods-12-00455-s001.zip › foods-2134004-supplementary.pdf]

foods

MDPI

Supplementary Materials

Table S1. Copy number associated with the GE rice line in the samples n°1-19. Each sample was tested in triplicate using a previously developed 2-plex ddPCR method targeting the GE rice line [16]. For each sample, the average values of the 3 replicates and the associated standard deviation are indicated.

Sample n°	Sample types	Sample description	GE rice line – Copy number	References
**1**	Rice seeds	GE rice line 100% - Parental rice line 0%	13,733.3 ± 141.9	[16]
**2**		GE rice line 99.9% - Parental rice line 0.1%	13,346.7 ± 213.8	[16]
**3**		GE rice line 99.1% - Parental rice line 0.9%	13,273.3 ± 253.2	[16]
**4**		GE rice line 95% - Parental rice line 5%	12,113.3 ± 375.4	[16]
**5**		GE rice line 90% - Parental rice line 10%	10,753.3 ± 75.7	[16]
**6**		GE rice line 50% - Parental rice line 50%	5,640.0 ± 87.2	[16]
**7**		GE rice line 10% - Parental rice line 90%	1,153.3 ± 33.2	[16]
**8**		GE rice line 5% - Parental rice line 95%	528.0 ± 13.1	[16]
**9**		GE rice line 0.9% - Parental rice line 99.1%	85.3 ± 8.3	[16]
**10**		GE rice line 0.1% - Parental rice line 99.9%	10.8 ± 1.5	[16]
**11**		GE rice line 0% - Parental rice line 100%	0 ± 0.0	[16]
**12**	Rice noodles	GE rice line 100% - Parental rice line 0%	13,610.0 ± 190.8	This study
**13**		GE rice line 99.9% - Parental rice line 0.1%	13,596.4 ± 213.9	This study
**14**		GE rice line 99.1% - Parental rice line 0.9%	13,487.0 ± 311.8	This study
**15**		GE rice line 0.9% - Parental rice line 99.1%	122.5 ± 9.5	This study
**16**		GE rice line 0.1% - Parental rice line 99.9%	13.6 ± 5.7	This study
**17**		GE rice line 0% - Parental rice line 100%	0 ± 0.0	This study
**18**	Crop mixtures	GE rice line 0.1% - Parental rice line 0.1% - WT maize 99.8%	10.6 ± 1.6	[16]
**19**		GE rice line 0.1% - Parental rice line 0.1% - WT 99.8%	9.3 ± 2.8	[16]

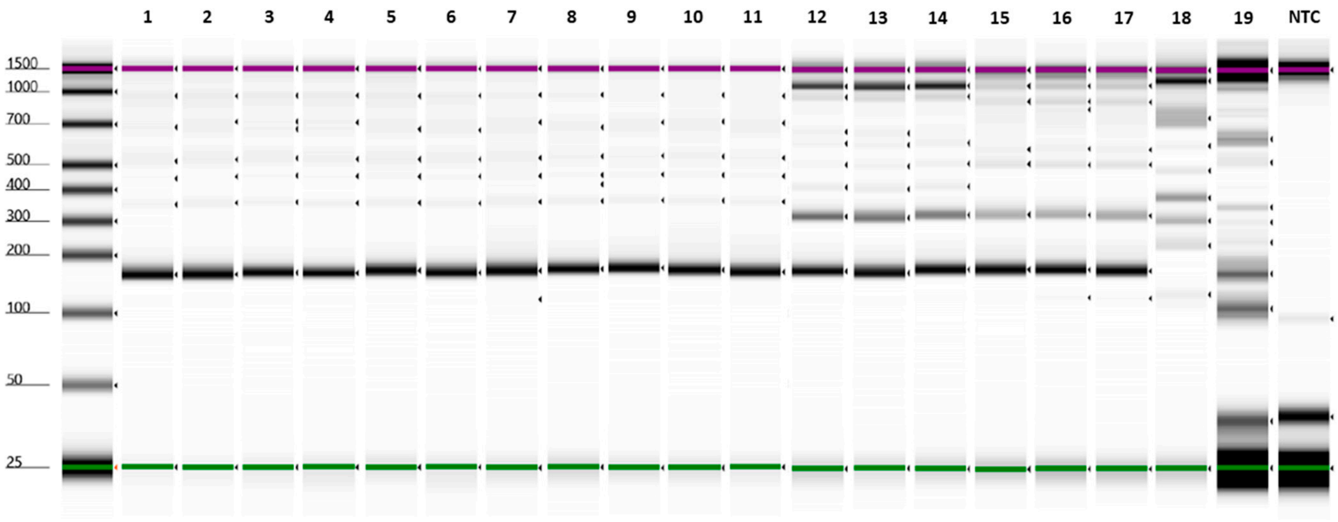

Figure S1. Visualization of final PCR products from samples n°1-19 and NTC (No Template Control).

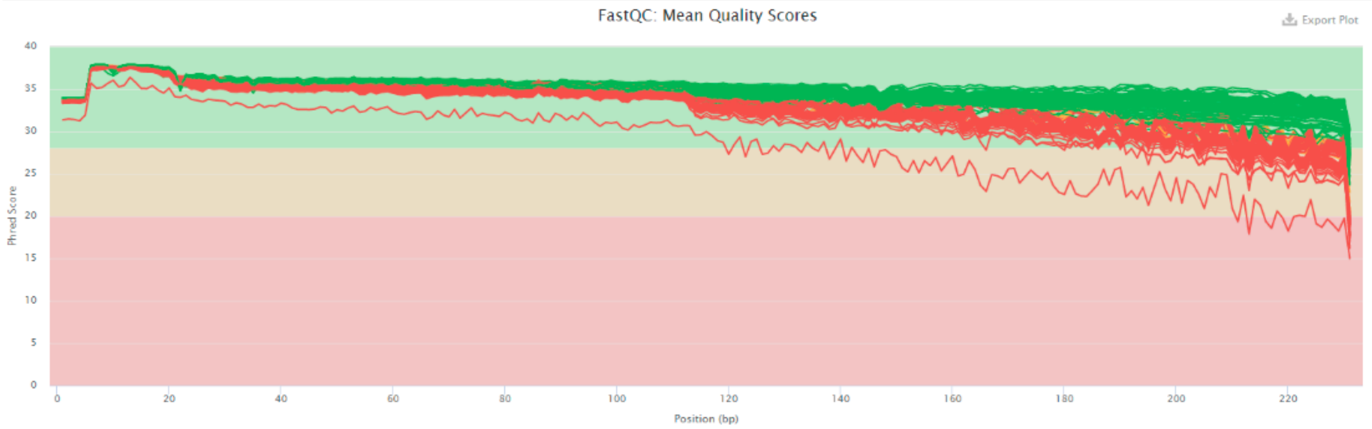

Figure S2. FastQC of the raw sequencing data.
